# In Vivo Regulation of Small Molecule Natural Products, Antioxidants, and Nutrients by OAT1 and OAT3

**DOI:** 10.3390/nu16142242

**Published:** 2024-07-12

**Authors:** Kian Falah, Patrick Zhang, Anisha K. Nigam, Koustav Maity, Geoffrey Chang, Jeffry C. Granados, Jeremiah D. Momper, Sanjay K. Nigam

**Affiliations:** 1 Department of Biology, University of California San Diego, La Jolla, CA 92093, USA; 2 Skaggs School of Pharmacy and Pharmaceutical Sciences, University of California San Diego, La Jolla, CA 92093, USA; 3 Department of Pharmacology, School of Medicine, University of California San Diego, La Jolla, CA 92093, USA; 4 Department of Bioengineering, University of California San Diego, La Jolla, CA 92093, USA; 5 Department of Pediatrics, University of California San Diego, La Jolla, CA 92093, USA; 6 Department of Medicine (Nephrology), University of California San Diego, La Jolla, CA 92093, USA

**Keywords:** transporters, vitamin, flavonoid, nutraceuticals, Ayurveda, plants, traditional Chinese medicine (TCM), chemoinformatics, machine learning, SLC22, organ crosstalk, phytochemicals

## Abstract

The organic anion transporters OAT1 (SLC22A6) and OAT3 (SLC22A8) are drug transporters that are expressed in the kidney, with well-established roles in the in vivo transport of drugs and endogenous metabolites. A comparatively unexplored potential function of these drug transporters is their contribution to the in vivo regulation of natural products (NPs) and their effects on endogenous metabolism. This is important for the evaluation of potential NP interactions with other compounds at the transporter site. Here, we have analyzed the NPs present in several well-established databases from Asian (Chinese, Indian Ayurvedic) and other traditions. Loss of OAT1 and OAT3 in murine knockouts caused serum alterations of many NPs, including flavonoids, vitamins, and indoles. OAT1- and OAT3-dependent NPs were largely separable based on a multivariate analysis of chemical properties. Direct binding to the transporter was confirmed using in vitro transport assays and protein binding assays. Our in vivo and in vitro results, considered in the context of previous data, demonstrate that OAT1 and OAT3 play a pivotal role in the handling of non-synthetic small molecule natural products, NP-derived antioxidants, phytochemicals, and nutrients (e.g., pantothenic acid, thiamine). As described by remote sensing and signaling theory, drug transporters help regulate redox states by meditating the movement of endogenous antioxidants and nutrients between organs and organisms. Our results demonstrate how dietary antioxidants and other NPs might feed into these inter-organ and inter-organismal pathways.

## 1. Introduction

Multi-specific drug transporters transport pharmaceuticals, endogenous metabolites, essential nutrients, antioxidants, and a wide range of other small molecules present in natural products (NPs) [1]. While much regulatory interest has focused on transporter-level drug–drug interactions (DDIs), it is increasingly recognized that many commonly ingested compounds and endogenous metabolites are also substrates, inhibitors, or inducers of the same transporters. These drug–metabolite (DMI) and drug–nutrient interactions (DNI) present major challenges [2].

DDIs, DMIs, DNIs, and drug–NP interactions at the level of transporters have the potential for adverse drug side effects or loss of efficacy, as normal disposition may be affected by another competing molecule. These types of interactions are not well understood, but many of the drugs, metabolites, nutrients, and phytochemicals are transported by drug transporters. Chronic use of the commonly prescribed diabetic medication metformin (transported by OCTs and MATEs) has been shown to affect levels of vitamin B12, vitamin D, and their metabolic byproducts [3]. While DDIs are increasingly discussed in the ADME and pharmacokinetics literature, DNIs and drug–NP interactions remain understudied but may have profound effects on transporters and DMEs [4]. Some examples of DNIs can be seen with commonly used pharmaceuticals. Ingestion of grapefruit juice is not advised when a patient is taking drugs that are CYP3A4 substrates, as some of the compounds found in grapefruit juice (e.g., bergamottin, naringenin, furanocoumarin) inhibit the activity of CYP3A4 and increase the oral bioavailability of the drug [5]. Nutrients and natural products cover a wide range of molecules, including the small organic molecules found in traditional Chinese medicines (TCM) and herbal teas [6,7]. These traditional herbs are taken to alleviate a variety of ailments [8,9].The popularity of these remedies and teas calls for a deeper investigation of DNIs at the site of drug transporters.

A network of 500–1000 genes is involved in the absorption, distribution, metabolism, and elimination (ADME) of exogenous and endogenous small molecules [10]. This network consists of SLC (solute carrier) and ABC transporters (ATP-binding cassette), DMEs (drug metabolizing enzymes), and transcriptional regulators (e.g., nuclear receptors).

According to remote sensing and signaling theory, this network is necessary for organ crosstalk and interorgan communication, including between the gut microbiome and host [11]. Within this ADME network is the SLC22 transporter family, which consists of multi-, oligo-, and mono-specific transporters. Some family members have received increased attention from drug regulatory agencies such as the U.S. Food and Drug Administration (FDA).

Two of the original transporters selected by the FDA to evaluate transporter-mediated interactions are OAT1 (SLC22A6) and OAT3 (SLC22A8). These are multi-specific transporters expressed in the proximal tubule of the kidney [12]. Their high expression on the basolateral side of the proximal tubule mediates kidney uptake and urinary clearance of certain compounds, making them key proteins in regulating the levels of numerous drugs and metabolites [13]. The diversity of OAT1 and OAT3 substrates range from pharmaceutical drugs (e.g., diuretics, antibiotics) to endogenous metabolites (e.g., prostaglandins) and commonly ingested compounds such as vitamins [14,15]. Due to the multi-specific nature of these two renal transporters, a range of small molecule natural products (NPs) can potentially compete with drugs and metabolites for access to the transporter, affecting their distribution and clearance.

In the near future, it seems likely that many other traditional medicine or natural products will increasingly be used for a variety of health conditions in clinics and hospitals. There is growing agreement that these compounds ought to be analyzed with the same scientific rigor as other drugs. The ADME properties of these NPs as they pertain to enzyme and transporter activity need to be fully probed, especially for multi-specific proteins such as drug transporters that have demonstrated a capacity to handle structurally diverse compounds with different origins and mechanisms of action.

Here, we focused on OAT1 and OAT3 transport/inhibition of natural products, most of them plant-derived. These NPs may have direct roles in the essential growth and development processes of plants or affect the animals ingesting the NPs, for example, by acting as cofactors for metabolic enzymes [16]. They may also play roles in diverse processes relating to pigmentation, ripeness, and aroma formation, as well as protecting against both herbivores and pathogens [17]. OAT1 and OAT3 have even been found to transport Ochratoxin A, a common fungal toxin found in food products from wheat to wine [18]. Small molecules within these NPs include organic acids, aldehydes, ketones, alcohols, esters, lactones, sulfur compounds, acetals, furans, phenols, terpenes, and epoxides [19].

Based on in vitro studies of these small molecules in NPs, OAT1 and OAT3 have been considered key for understanding how the body handles NPs. Flavonoids are polyphenols that are present as diverse mixtures in teas, legumes, nuts, leafy vegetables, and citrus fruits [20]. This means that a single serving of legumes could contain multiple types of flavonoids. These phytochemicals may enhance metabolic efficiency by preventing the buildup of free radicals [21]. Polyphenols interact strongly with OAT1 and have been reported to inhibit its activity in vitro. Epigallocatechin gallate (EGCG), another OAT1 substrate, is found in commonly ingested natural substances, such as green or black teas [22]. EGCG and related compounds appear to prevent excessive accumulation of free radicals but can potentially cause adverse effects by inhibiting OAT1 transport in the kidney [23]. These examples highlight the importance of understanding the interaction of natural products with OAT1 and OAT3. Direct toxicity is also a major concern. For instance, some NPs, such as the aristolochic acid present in certain grains and teas, are transported into the kidney by OAT3 and are toxic to the kidney [24].

Thus, there is tremendous interest in drug–NP interactions at the level of drug transporters like OAT1 and OAT3 and unexpected NP-related toxicity [2,25]. However, most of these studies, particularly in relation to OAT1 and OAT3, are based on in vitro data obtained using selected compounds from cells overexpressing the transporters. Nevertheless, it is now clear from multi-omics in vivo analyses of the OAT knockout mice that in vitro data may not be reflective of the in vivo situation, representing a biased selection. A clearer picture generally emerges from a combination of in vivo data in knockout animals supplemented by in vitro transport studies.

Here, we take such an approach, comparing the NPs altered in vivo in knockout (KO) to wildtype (WT) mice. Coupled with in vitro evidence of the interaction between these transporters and natural products (using transfected cells and a relatively novel binding assay [26]), we were able to define likely direct interactions between NPs and OAT1/OAT3 proteins. Chemoinformatic analyses further helped us to define the chemical classes of molecules in NPs that are regulated by OAT1 and OAT3. This work solidifies the important role that well-studied drug transporters have in the regulation of natural products and identifies a set of strategies that can be used to better understand the interactions of NPs, nutrients, and antioxidants with transporters in vivo.

## 2. Materials and Methods

### 2.1. Natural Product (NP) Databases

**Figure 1:**
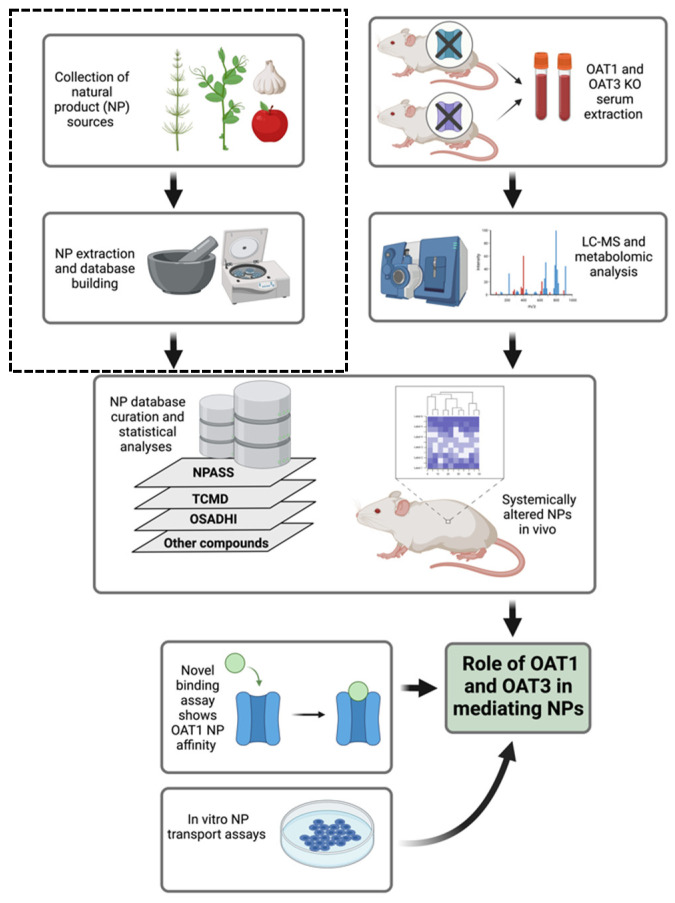
Schematic detailing the workflow of this study. Three databases were used to create a standard of natural products to filter out the relevant compounds from the in vivo and in vitro assays. The upper left graphics are meant to convey how databases are generally made by others. NPASS represents The Natural Product Activity and Species Source. TCMD represents The Traditional Chinese Medicine Database. OSADHI represents the Online Structural and Analytics-Based Database for Herbs of India. In vivo serum metabolomics were conducted on Oat1 and Oat3 KO mice compared to WT controls, and hundreds of compounds were found to be altered under each knockout condition. In vitro assays were used to further screen for natural products of interest. A cell-based transport assay was conducted for HEK293 cells overexpressing human OAT1 protein. An OAT1 protein model was also used to detect direct binding of natural products against a standardized substrate. Figure created using BioRender.com.

Three different NP databases were analyzed. They contained information from well-established traditional medicines and other compounds found in plants, bacteria, and fungi. These databases are described in more detail in the Results section. They include The Natural Product Activity and Species Source (NPASS) database [27]; the Traditional Chinese Medicine Database (TCMD) [28]; and the Online Structural and Analytics-Based Database for Herbs of India (OSADHI) [29]. The three NP databases were employed to generate a list of known OAT1 and OAT3 substrates that were considered natural products. The workflow for these and other methods is depicted in Figure 1.

### 2.2. Animals

Mice were group-housed with up to five mice per cage under a 12 h light–dark cycle and were provided access to food and water ad libitum. All the experiments were performed on female age-matched (within 2 weeks) mice that were between 12 and 20 weeks of age. The experimental protocols were in accordance with the National Research Council guidelines [30] and were approved by the Institutional Animal Care and Use Committee at UC San Diego. Oat1 KO and Oat3 KO mice were generated and maintained as previously described [31,32].

### 2.3. Metabolomics Serum Collection

Serum samples from adult female wild-type (WT) control, Oat1 KO, and Oat3 KO, mice were obtained and stored at −80 °C. All the samples were shipped overnight in dry ice to Metabolon (Durham, NC, USA) for metabolomic analyses [33]. The samples were first prepared using the MicroLab STAR^®^ system (Hamilton, Reno, NV, USA) and subjected to ultra-high-performance liquid chromatography–tandem mass spectroscopy (UPLC-MS/MS) as previously described [34].

### 2.4. Metabolomics, Pathway Analyses, Chemoinformatics, and Data Visualization

This study included a novel set of serum metabolomics for female Oat1 and female Oat3 knockout mice, detecting over 1000 metabolites in each panel. These results were used to assess the in vivo evidence linking NPs across the 3 NP databases to metabolites analyzed in the knockout mice. Each metabolomics study was first tested to ensure a clear separation between all the detected metabolites in the wildtype and knockout conditions. Following quantile normalization, log transformation, and pareto scaling of all the original metabolomics counts, a partial least squares discriminant analysis was conducted to generate a 2D scores plot. Enrichment scores were calculated for all Metabolon subpathways using the following formula:

Enrichment score = (k/m)/((n − k)/(N − m)), where m = the number of metabolites in the pathway, k = the number of significant metabolites in the pathway, n = the total number of significant metabolites, and N = the total number of metabolites. Generally, a *p*-value of <0.05 was used as the criteria for significance (see Supplementary Table S1).

The molecular properties of the compounds were calculated using the chemoinformatics program RDKit [35]. Using the RDKIT Cheminformatics Toolbox, ~200 molecular descriptors were generated as features for each natural product match. After removing highly correlated properties and performing a dimensionality reduction to determine the most important properties, several were selected for further analyses based on their ease of interpretability. Freeviz is a method for multivariate analysis in the Orange Data Mining environment [36].

### 2.5. In Vitro Inhibition Uptake Assay

Human embryonic kidney (HEK) cells were transfected with human OAT1 (SOLVO Biotechnology) to overexpress the transporter. The cells were maintained in DMEM (Thermo Fisher Scientific, Waltham, MA, USA, catalog 11965092) supplemented with 10% FBS (Thermo Fisher Scientific, catalog 26140079), 1% penicillin/streptomycin (Thermo Fisher Scientific, catalog 15140122), and blasticidin (InvivoGen, San Diego, CA, USA, catalog ant-bl-1), a selective marker for OAT1 expression. Once confluent cells were tested for mycoplasma infection. Trypsin (Thermo Fisher Scientific, catalog 25200056) was then used to detach the cells, which were then plated on 96-well plates in media without blasticidin and grown until confluent, typically around 24 h. Natural products were added at 2 mM, and a serial dilution was performed across the next 9 columns. Probenecid was used as a standard inhibitor to compare the strength of inhibition. A standard concentration of 10 μM 6-carboxyfluorescin (6-CF) was added to the plate for 10 min to allow for cellular uptake of this standard OAT1 substrate. The cells were rinsed with ice-cold PBS 3 times, with the final wash remaining in the well. Fluorescence was measured using a FilterMax F5 multi-mode plate reader (Molecular Devices) with excitation at 485 nm and detection at 535 nm.

In addition to our in vitro work, data from our lab and from other labs were collected to compare to the in vivo metabolomics analysis results (see Table 1). These inhibition uptake assays generally used HEK293 or LLC-PK1 kidney cell lines transfected with OAT1. Please see Table 1 and the Results section for details. Standard OAT1 substrates, para-aminohippuric acid (PAH) or 6-carboxyfluorescein (6-CF), were typically used to establish baseline uptake, which allowed for the determination of the potential OAT1-suppressive effect of each NP tested.

### 2.6. OAT1 Magnetic Bead Binding Assay

An OAT1–3C–GFP complex was generated to bind to magnetic beads in an in vitro substrate binding assay. The details of this assay have been described previously [10]. 6-CF was used as a standard OAT1 substrate to compare the binding ability of competing test substrates. The standard level of fluorescence of only 6-CF binding the OAT1 protein–bead complex was compared to the shift in fluorescence when the potential new OAT1 substrates were introduced. A normalized downshift of −0.05 from the control fluorescence was determined to be significant. Natural products of interest were screened and categorized as an OAT1 binder or nonbinder based on their competition against 6 μM 6-CF.

### 2.7. ChemRICH Analysis

ChemRICH is a chemical similarity analysis tool that uses clinically relevant pathway designations in tandem with Tanimoto substructure chemical similarity coefficients to cluster metabolites into non-overlapping chemical groups. Calculations for the statistical significance of these clusters were obtained using self-contained Kolmogorov–Smirnov tests [37]. ChemRICH was utilized to perform similarity enrichment calculations for significantly altered metabolites and provide alternative pathway designations based on chemical similarity as well as ontology.

## 3. Results

### 3.1. Serum Metabolomics of Oat1 and Oat3 KO Mice Reveal Significant Alterations in Circulating Levels of Natural Products

Mouse serum metabolomics data allowed us to analyze the global metabolic changes in female Oat1 and Oat3 KO models compared to WT mice in two separate studies. Serum was collected from Oat1 (n = 4) and Oat3 (n = 6) KO mice, then compared to WT (n = 4 and n = 6, respectively) mice. We determined whether a compound was a natural product by matching the measured compounds to entries in three different databases pertaining to NPs, individual phytochemicals, and medicinal plants. The Natural Product Activity and Species Source (NPASS) database contains over 35,000 natural products from plants, bacteria, animals, and even fungi [27]. For traditional Chinese medicines, we utilized the Traditional Chinese Medicine Database (TCMD), which contains over 20,000 plant-derived compounds and herbal medicines utilized for treating ailments across the globe [28]. The third database used to collect NPs of interest was the Online Structural and Analytics-Based Database for Herbs of India (OSADHI), with over 27,000 unique phytochemicals and medicinal plants [29]. Together, these three NP databases were used to create a list that determined what OAT1 and OAT3 substrates were deemed “natural products” (for the purpose of our subsequent analysis of NPs altered by the loss of OAT1 or OAT3 in vivo) (Figure 1). This analysis is unique from previous OAT1 and OAT3 investigations of these and other datasets, as it examines a subset of NPs and nutrients that are commonly ingested in the diet and present in the aforementioned NP databases. Other work has focused on all metabolites captured and/or specific biochemical pathways, emphasizing systemic physiology such as lipid and tryptophan metabolism [34,38].

OAT1 metabolomics revealed a total of 282 compound matches across all 3 NP databases, with 30 significantly altered unique compounds that matched at least one database (Supplementary Table S1). There were 17, 14, and 16 significant matches to the NPASS, TCMD, and OSADHI databases, respectively. OAT3 metabolomics had a total of 274 compound matches across each NP database, with 46 significantly altered unique compounds that matched at least one database (Supplementary Table S2). There were 25, 27, and 28 significant matches to the NPASS, TCMD, and OSADHI databases, respectively.

### 3.2. Partial Least Squares Discriminant Analysis Yields Clear Separation of Natural Products in Oat1 and Oat3 KO Serum

**Figure 2:**
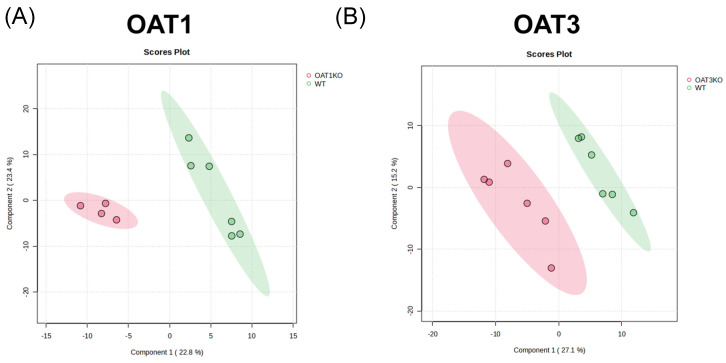
Separation of serum metabolomics of Oat1 KO and Oat3 KO mice for all natural product matches revealed by partial least squares discriminant analysis (PLSDA). (**A**) Following quantile normalization, log transformation, and pareto scaling of all the matched metabolite counts, separation of the Oat1 KO and WT natural product database matches was determined via PLSDA. There was a clear separation between the knockout and wildtype mice against database-matched natural products. (**B**) An identical analysis was applied to the Oat3 KO metabolomics study. There was a clear separation of the knockout and wildtype natural products across each genotype.

**Figure 3:**
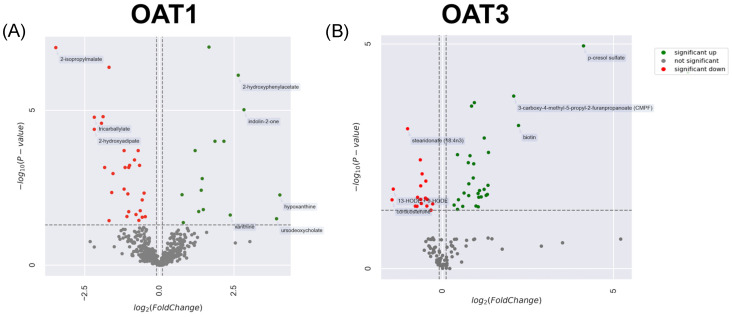
Elevated and decreased natural products in both Oat1 KO and Oat3 KO mice. (**A**) A volcano plot is included for all natural product matches that were significantly altered by Oat1 KO (30). (**B**) A volcano plot is included for all natural product matches that were significantly altered by Oat3 KO (46). Green dots indicate significantly elevated metabolites in KO serum (*p* < 0.05). Red dots indicate significantly decreased metabolites in serum. Asymptotic vertical lines are at fold changes of −0.9 and 1.1.

There was a clear separation of KO vs. WT data when focusing on our list of 76 OAT1/3-associated NPs present in the in vivo metabolomics data from both knockouts. A partial least squares discriminant analysis (PLSDA) was conducted, and the top two principal components explained more than 45% of the variance for the Oat1 KO vs. WT natural product space (Figure 2A). An analysis of the Oat3 KO space also showed separation, but with a greater variance across the knockout space. The first two principal components explained 42% of the variance (Figure 2B). Across the NP matches in the Oat1 KO chemical spaces, there were many significantly elevated and decreased (*p* ≤ 0.05) compounds (Figure 3). In the Oat1 and Oat3 KO mice, where there is a loss of kidney transporter function, previous studies have revealed that compounds that are substrates of these transporters are also elevated in the bloodstream [26,39]. Many of these compounds are also altered when humans are treated with the OAT-inhibiting drug probenecid [40].

### 3.3. Cofactors and Vitamins Are among the Several Pathways Altered in Oat3 KO Serum

**Figure 4:**
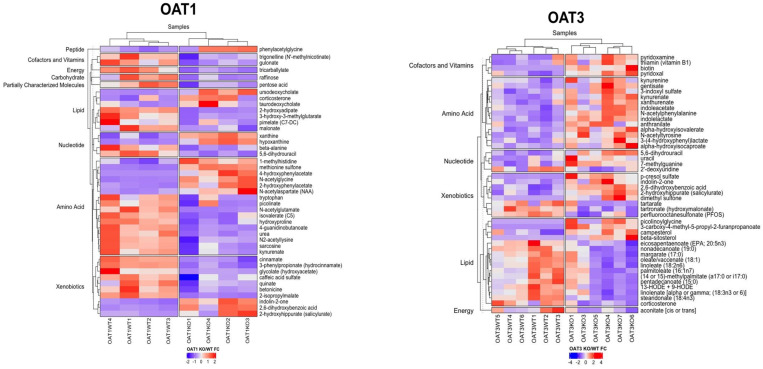
Natural product matches for OAT1 and OAT3 mice fluctuated across various metabolon superpathway designations. These heatmaps display the metabolites from the natural product matches and are clustered across rows via metabolon-designated superpathways. Column dendrogram clustering revealed relatively clear differences between the knockout and wildtype samples for both OAT1 and OAT3, indicating that NPs are likely mediated by OATs in vivo.

A superpathway enrichment score analysis revealed that the Cofactor and Vitamins superpathway was the most enriched superpathway in the Oat3 KO serum. Indeed, its enrichment score was nearly double that of the next most implicated superpathway: amino acid metabolism (Figure 4). Looking specifically into the subpathways within cofactors and vitamins, we see that biotin (vitamin B7) metabolism, vitamin B6 metabolism, and thiamine (vitamin B1) metabolism were the three most enriched subpathways in Oat3 KO serum. 

**Figure 5:**
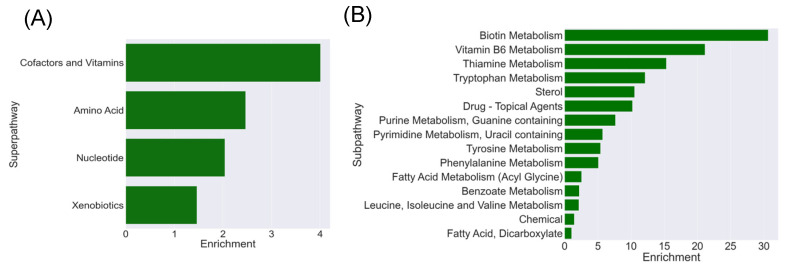
Loss of OAT3 in vivo alters many metabolic pathways containing natural products. (**A**) Cofactors and vitamins were the most enriched increased superpathways among elevated Oat3 KO in vivo natural products matched to the combined databases; nearly double the enrichment score of the next closest amino acid pathway. Nucleotide and xenobiotics were the final two superpathways enriched in the knockout mice. (**B**) Enrichment plots sorted based on the associated subpathways with at least 1 significantly elevated compound. Cofactors and vitamins were the most enriched increased superpathway; this contained the subpathways of biotin (vitamin B7), vitamin B6, and thiamine (vitamin B1) metabolism, which were the 3 most enriched subpathways for elevated compounds.

More specifically, our Oat3 KO mice showed a significant elevation of biotin (*p* ≤ 0.05, fold-change: 4.55). Biotin, also known as vitamin B7, plays important roles in controlling metabolic homeostasis. In addition to acting as a coenzyme for gluconeogenesis and amino acid and fatty acid metabolism, biotin seems to play a role in the transcriptional regulation of certain proteins via modification of histones such as stimulation of glucokinase [41]. Vitamin B6 is another important coenzyme in biosynthetic and catabolic reactions. It exists as either pyridoxic acid, pyridoxine, pyridoxal, or pyridoxamine. Natural sources of vitamin B6 include potatoes, bananas, nuts, and animal products such as meat and eggs; though it is also synthesized de novo by the gut microbiome [42]. Two forms of vitamin B6, pyridoxal (*p* ≤ 0.005, fold-change: 1.86) and pyridoxamine (*p* ≤ 0.05, fold-change: 2.32), were significantly elevated in the Oat3 KO serum (Figure 5). Furthermore, vitamin B6 has recently been shown to be a potential endogenous biomarker of OAT1/3 transport activity. A study of humans treated with probenecid, a potent OAT1/3 inhibitor, revealed elevated pyridoxic acid levels in the blood [40]. Our knockout murine metabolomics, in tandem with previous probenecid OAT3 inhibition studies, suggest that vitamin B6 and its downstream metabolites may be substrates of OAT3.

Thiamine, or vitamin B1, plays a role in controlling the metabolism of glucose, fatty acids, and amino acids to provide energy for the nervous system. Thiamine is additionally important in maintaining nerve function through the production of myelin, and it acts as a cofactor to generate neurotransmitters such as acetylcholine and serotonin [43]. In the serum of Oat3 KO mice, we found that thiamine was significantly elevated (*p* ≤ 0.05, fold-change: 2.06). It is interesting that OAT3 regulates the levels of many B vitamins in vivo, despite diverse structures. This suggests some connection between OAT3 and vitamin levels and other associated metabolic pathways.

### 3.4. Chemical Property Analysis Helps Differentiate OAT- and OAT3-Dependent Natural Products

**Figure 6:**
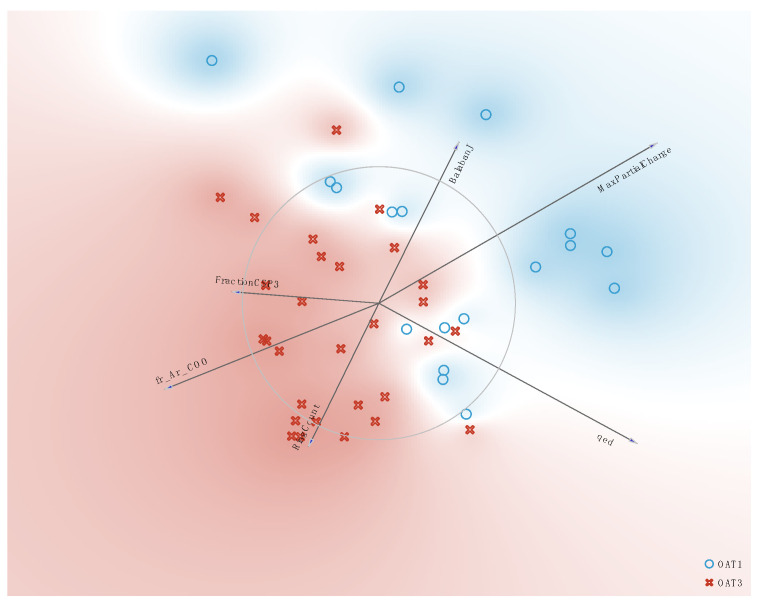
Freeviz Diagram illustrating the impact of various molecular properties on the separation of OAT1- and OAT3-interacting NPs. Freeviz is a multivariate analysis tool in Orange [36]. Shown here is the influence of six molecular properties (based on chemoinformatic analysis using RDKit) on whether the NPs in the dataset are affected by the knockout of Oat1 or Oat3 in vivo.

To better understand the chemical properties of the natural products that were altered in the two knockouts, we took a chemoinformatic approach to identify the molecular properties based on the NP structures. More than a hundred molecular properties of NPs altered in the Oat1 KO and Oat3 KO serum were calculated using RDKit [35]. A subset of these appeared useful in distinguishing OAT1- vs. OAT3-dependent NPs in vivo. Some of the most influential of the molecular properties are shown in the Freeviz diagram (Figure 6), a multivariate analysis tool in the Orange environment [36].

### 3.5. OAT1 In Vitro Uptake Assay and Protein Binding Validation

**Figure 7:**
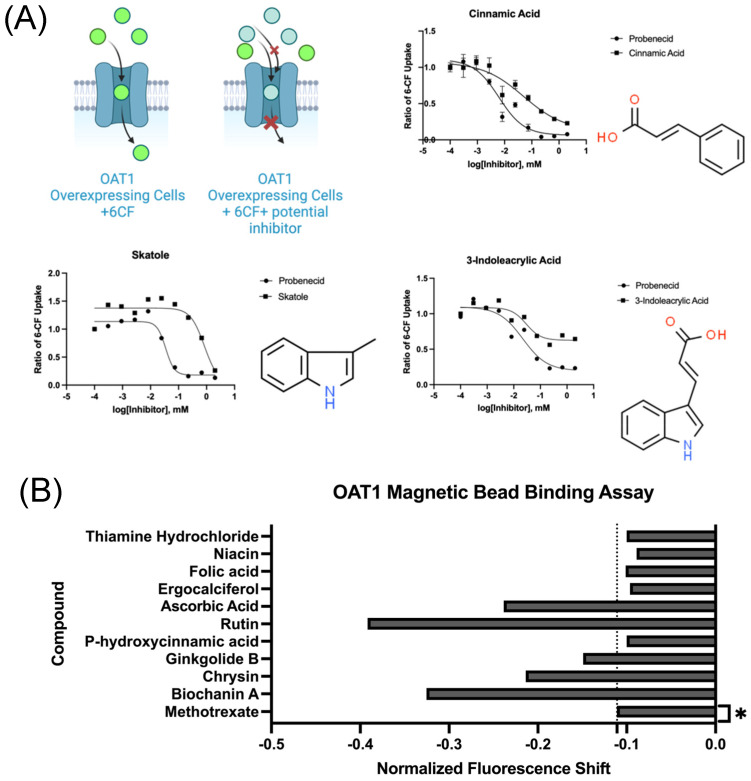
In vitro confirmation of OAT1-interacting natural product matches. (**A**) Human embryonic kidney cells overexpressing OAT1 transporters were screened using an uptake inhibition assay of fluorescent OAT1 substrate 6-carboxyfluorescin. These were screened against different concentrations of either a known OAT1 inhibitor (probenecid) or the compound of interest. (**B**) An OAT1 protein binding assay was used to screen natural products of interest. We utilized a magnetic bead binding assay against 6-carboxyfluorescence to screen a set of natural products. This was used to determine compounds that caused a significant shift in fluorescence, revealing a direct interaction between OAT1 and these selected vitamins and plant-derived compounds. * Methotrexate is an established OAT substrate and is used here to compare the relative affinity for natural products, revealing how certain natural products such as biochanin A and chrysin exhibited far greater binding strength to OAT1 than this pharmaceutical.

A combination of in vitro methods was used to identify natural products interacting with OAT1. A standard in vitro assay with HEK293 cells overexpressing OAT1 was conducted to confirm some expected natural product inhibitors of the transporter (Figure 7A). In addition to this, a ligand binding assay using OAT1 protein was employed to screen sets of natural products for direct interaction with the protein binding. Vitamins and cofactors (e.g., ascorbic acid) were shown to have significant OAT1 binding activity in addition to plant-derived flavonoids such as biochanin A, ginkgolide, and chrysin (Figure 7B). Our data from these in vitro assays were used to supplement and validate some previous in vitro analyses of NPs shown to be associated with OAT1 (Table 1). We were able to generate a list of 47 NPs interacting with OAT1 in vitro after screening for matches across the three NP databases (Supplementary Table S3).

**Table 1:** List of OAT1 in vitro assay-associated natural products from our studies and previous work conducted by our lab and others. For previous work, the original papers can be found in the bibliography according to their references. * Previous publications from this lab.

	Binding Assay	In Vitro Cell-Based Transport Assay	Previous in Vitro Cell Assay Validation	References
Citric acid	**X**			
Alpha ketoglutaric acid	**X**			
Succinic acid	**X**			
D-+-Malic acid	**X**			
Elaidic acid	**X**			
Cis-aconitic acid	**X**			
Arabinose	**X**			
Rutin	**X**			
Biochanin A	**X**		**X**	[44]
Chrysin	**X**		**X**	[44]
Ginkgolide B	**X**		**X**	[44]
P-hydroxycinnamic acid	**X**			
Serotonin	**X**			
N-acetylglycine	**X**			
Homovanillic acid	**X**			
Saccharin	**X**			
Ascorbic acid	**X**			
Folic acid	**X**			
Thiamine hydrochloride	**X**			
Ergocalciferol	**X**			
Niacin	**X**	**X**		
Skatole		**X**		
3-Indoleacrylic acid		**X**		
Fisetin			**X**	[45]
Galangin			**X**	[45]
Luteolin			**X**	[45]
Morin			**X**	[45]
Myricetin			**X**	[44]
Silymarin			**X**	[44]
Diosmin			**X**	[44]
Genistein			**X**	[44]
Quercetin			**X**	[44]
Phloridzin			**X**	[44]
Lithospermic acid			**X**	[46]
Rosmarinic acid			**X**	[47]
Salvianolic acid A			**X**	[47]
Rhein			**X**	[47]
Wogonin			**X**	[48]
Baicalein			**X**	[48]
Aristolochic acid (AA-I)			**X**	[49]
Aristolochic acid (AA-II)			**X**	[49]
Gallic acid			**X**	[50]
Ferulic acid			**X**	[50]
Protocatechuic acid			**X**	[50]
Sinapinic acid			**X**	[50]
Vanillic acid			**X**	[50]
1,3-dicaffeoylquinic acid			**X**	[50]
18β-Glycyrrhetinic acid			**X**	[50]
Silybin			**X**	[51]
Emodin			**X**	[52]
Aloe emodin			**X**	[52]
Apigenin			**X**	[53]
Chrysophanol			**X**	[54]
Obtusifolin			**X**	[54]
Quercetin-3-O-glucuronide			**X**	[55]
Ellagic acid			**X**	[56]
Flavone			**X**	[57]
5,6,2′,6′-tetramethoxyflavone			**X**	[57]
Wedelolactone			**X**	[58]
Calycosin			**X**	[58]
Oroxylin A			**X**	[58]
Viscidulin III			**X**	[58]
Scullcapflavone II			**X**	[58]
Ginkgolide A			**X**	[59]
Bilobalide			**X**	[59]
Neochamaejasmine A			**X**	[60]
(−)-epigallocatechin-3-gallate (EGCG)			**X**	[23]
Cinnamic acid		**X**	X *	[26]
Indoxyl sulfate potassium salt			X *	[26]
4-Hydroxyphenylpyruvic acid			X *	[26]
4-hydroxyphenylacetic acid			X *	[26]
3-indoacetic acid			X *	[26]
2-oxindole			X *	[26]
Tyramine			X *	[26]
Indole-3-carboxaldehyde			X *	[26]

### 3.6. ChemRICH Classification and Analysis of OAT1 and OAT3 In Vitro Interacting Natural Products

Both knockout mice had significant alterations in natural products that mapped to the amino acid, nucleotide, lipid, and xenobiotic superpathways designated by the metabolon pathways. Most of the amino acids and xenobiotics (a broad metabolon grouping including many NPs) that were significantly altered were elevated in the bloodstream of the knockout mice (Figure 4 and Figure 5). Essential amino acids such as tryptophan and its metabolites, lipids, carboxylic acid byproducts, uric acid-associated compounds, gut microbiome-derived products, and other downstream compounds have, in some cases, been previously studied [26,34,38]. To better understand the compounds affected, chemical property analyses and chemical pathway mapping were utilized to study group trends.

**Figure 8:**
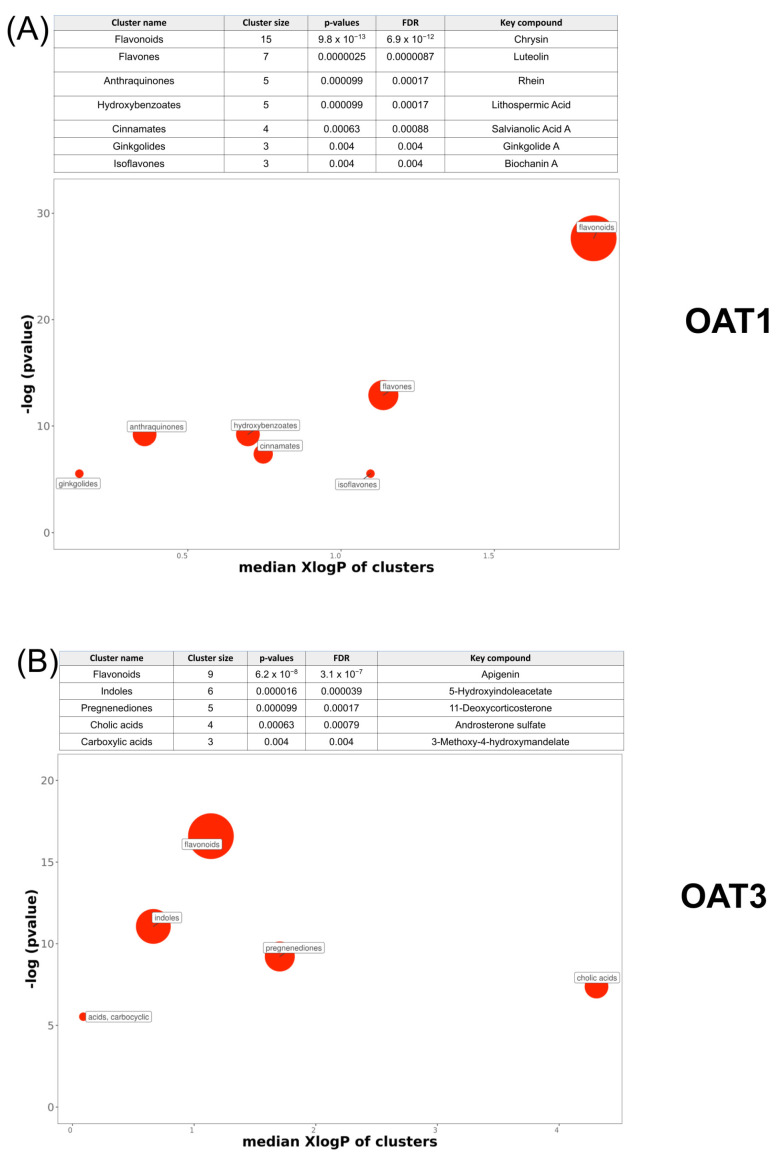
Clustering via ChemRICH chemical analysis of OAT1 and OAT3 in vitro natural products reveals clustering into specific biochemical groupings for each transporter, with an overlap of the flavonoids associated with each transporter. Clusters were determined for each compound using a combination of chemical property analysis and ontology mapping. Each circle reflects a significantly altered cluster of metabolites. The circle sizes reflect the total number of metabolites in each cluster set. The *X*-axis separates clusters in terms of their median cluster lipophilicity (XlogP-octanol/water partition coefficient). On the *Y*-axis, the enrichment *p*-values are given by the Kolmogorov–Smirnov test. (**A**) In addition to the general flavonoid group with 15 significantly altered NPs, the most enriched clusters in OAT1 were determined to be flavones, anthraquinones, hydroxybenzoates, and cinnamates, with a minimum of 5 in vitro-associated compounds. (**B**) For OAT3 matches, flavonoids, flavones, anthraquinones, hydroxybenzoates, and cinnamates were the most enriched clusters, with a minimum of 5 in vitro-associated compounds for each cluster.

Most of the identified natural products have an associated chemical structure that can further inform our metabolomics studies. By analyzing the chemical properties (e.g., molecular weight, solubility, etc.), we may be able to reveal a deeper chemical basis for transporter-natural product interactions. Therefore, we then performed a structure-based analysis to assign each compound to specific metabolite clusters. This was performed using ChemRICH—a computational tool that can be considered an alternative method for biochemical pathway mapping, emphasizing structural analysis and chemical ontologies. It combines this analysis with an enrichment test based on the Kolmogorov–Smirnov test [37]. Compared to traditional pathway mapping using databases like KEGG, ChemRICH generates non-overlapping sets of compounds and is not reliant on manual calls that can vary across databases. The plots of the OAT1- and OAT3-associated NPs against ChemRICH enrichment are shown in a plot of chemical properties such as the median XLogP (measure of hydrophobicity over hydrophilicity) versus the −Log (*p*-value) (Figure 8).

#### 3.6.1. OAT1 Chemrich Analysis

The ChemRICH analysis of these compounds matched 42 of the 47 NPs interacting with OAT1 in vitro to significant chemical clusters. This revealed seven unique chemical clusters. In addition to the general flavonoid class consisting of 15 compounds, flavones and isoflavones were also identified with 7 and 3 associated compounds, respectively. Some of these compounds are found in herbal remedies. For example, danshen is a root derived from red sage plants; it contains a diverse set of flavonoids and has been used as an herbal remedy for circulatory issues such as cardiovascular and cerebrovascular disease [61]. Its main therapeutic compounds include lithospermic acid, rosmarinic acid, and salvianolic acid A. Each of these three are in vitro inhibitors of the OAT1 transporter in cell-based uptake assays. As these compounds were from the same source extract, this indicates the complexity of potential transporter-level NP–drug interactions with flavonoids derived from commonly used herbal medicines.

The other generated classes and the associated number of compounds were hydroxybenzoates (5), anthraquinones (5), cinnamates (4), and ginkgolides (3) (Figure 8A). The anthraquinone cluster’s central associated compound was rhein. This is a common component of the medicinal root rheum, which is widely used as a naturopathic treatment for its anti-inflammatory and antipyretic properties in non-Western medicines [62]. An especially important consideration for rhein use is that 80% of its clearance relies on the glucuronidation or sulfation of rhein in the liver to be targeted for renal excretion [63]. Rhein has been shown to inhibit standard OAT1 activity in vitro, making it an important target for studying its potentially adverse effects on OAT transporters—especially considering the number of prescription anti-inflammatory and immunosuppressive agents that interact with OATs. For example, the drug methotrexate is known to inhibit OAT1/3 activity in vivo and in vitro [64]. Taken together, our ChemRICH analysis of the in vitro data revealed the importance of OAT interaction with flavonoids and associated subclasses, given that 25 compounds fell into a flavonoid-associated class.

#### 3.6.2. OAT3 Chemrich Analysis

Using previously generated in vitro data for compounds shown to be associated with OAT3, we screened these in vitro-validated compounds against the three natural product databases to generate a list of 41 natural products (Supplementary Table S4). A ChemRICH analysis of these compounds matched 27 of these compounds to significant chemical clusters. This revealed five unique classes of compounds associated with OAT3 in vitro: flavonoids, indoles, pregnenediones, colic acids, and carboxylic acids. Respectively, these chemical classes yielded cluster sizes of 9, 6, 5, 4, and 3 NPs (Figure 8B). Flavonoids were the largest class of OAT3-associated compounds, with nine flavonoids found from the in vitro matches and apigenin being the key compound. The relevance of this NP family has been mentioned previously. The second-largest cluster was indoles, which may have similar anticancer and antibacterial properties to that of flavonoids but are structurally different [65].

## 4. Discussion

Through their activity at the basolateral surface of the proximal tubule of the kidney, OAT1 and OAT3 are responsible for maintaining homeostasis and appropriate renal clearance of diverse metabolites, drugs, toxins, antioxidants, and signaling molecules [66]. Their substrates also include natural products, antioxidants, vitamins, and nutrients, which serve important roles in metabolism and cell signaling [67,68]. OAT1 and OAT3 also handle a wide variety of phytochemicals found in the NP databases we analyzed. These natural products have the potential to interfere with drug metabolism and/or drug transport. Likewise, there is potential for metabolite–NP interactions at the transporter level.

Although plants are important sources of exogenous compounds that are vital to bodily functions, uncontrolled levels of these molecules and their byproducts can be associated with adverse effects. Many plants, such as kale, spinach, and rhubarb, provide nutrients such as vitamin A, multiple B vitamins, and vitamin C and are thought to promote health through their high fiber levels and other effects. However, certain compounds derived from these plants can be toxic at high levels. Oxalate is present in many of these leafy greens such as spinach or kale and is even found in other sources such as fruit and nuts. Excess oxalate intake has been shown to cause the formation of kidney stones at excessively high levels [69]. The clearance of many potentially toxic organic anions, derived from common dietary sources that are otherwise beneficial, occurs partly or largely via OAT1 and OAT3 [70].

Flavonoids are an abundant secondary metabolite of plants that can be found in fruits, vegetables, grains, flowers, tea, and wine [71]. Current and previous work by our lab and others has revealed several flavonoids that strongly interact with OAT1 transporters. The flavonoids that inhibit standard OAT1 activity in vitro include derivatives of genistein, daidzein, quercetin, and epigallocatechin gallate [44]. Some of these compounds are also transported by OATs either in their sulfated or glucuronidated forms, following conjugation in the liver [45,72]. Phase II metabolism generates compounds with increased hydrophilicity to allow for more efficient excretion. The sulfated forms of flavonoids exhibit stronger interactions with the OAT1 transporter and can potentially limit the uptake of other OAT1 substrates.

A ChemRICH analysis of in vitro literature revealed that the NP family of flavonoids were the most broadly interacting NPs with OAT1 and OAT3 by in vitro assays, including those performed in previous studies by our lab [38]. Flavonoids are among the most commonly consumed natural product classes, being found in sources such as tea, wine, fruits, grains, and vegetables [73]. The flavonoid class can be further broken down into six subclasses (e.g., isoflavonoids, flavanols, flavones, flavanones, chalcones, and anthocyanins). Within these subclasses are multiple well-studied flavonoids that may be therapeutically useful by inhibiting lipid peroxidation and enhancing antioxidative ability. They have received attention in a wide variety of disease contexts, including cancer, atherosclerosis, diabetes, hepatotoxicity, and heart disease [71,74,75].

Among the best-studied flavonoids are quercetin, luteolin, apigenin, and catechins, which have all demonstrated significant interactions with OAT1 and OAT3 in vitro [39,44,55]. There are also important interactions between glucuronidated flavonoids, particularly for OAT3 (Supplementary Tables S1–S4). Quercetin has been shown to exhibit cytotoxic effects within leukemic and breast cancer cells while also inducing S phase arrest [76]. Similarly, the possible anticancer activity of luteolin and apigenin has been demonstrated in vitro, where these NPs have suppressed proliferation of multiple cancer cell lines and downregulated tumorigenic pathways [77,78,79]. These NPs may also affect the progression of other diseases like diabetes, atherosclerosis, and hypertension. These NPs are often found in various fruits and green vegetables, but luteolin is commonly derived from chrysanthemum leaves, and apigenin is also found in chamomile leaves [80,81]. The perceived value of NPs in various disease states has been met with increased self-medication and over-the-counter use. With studies demonstrating that these commonly ingested flavonoids share specificity with OAT1 and OAT3, and thus the body’s main renal drug elimination routes, it is critical that transporter-level interactions between drugs and NPs be studied to prevent harmful DNIs and DNPIs.

For example, the interaction of antiviral drugs such as adefovir with OAT1 is inhibited in vitro by sulfated quercetin and genistein, which are OAT1 substrates in both their conjugated and original forms [44]. This interaction could conceivably prevent typical nephrotoxicity when adefovir would otherwise accumulate in the kidney via OAT1 uptake. This may be an example of a positive benefit of a DNI [55]. However, this same interaction could extend the half-life of drugs in the bloodstream, which may lead to adverse drug reactions. Although such studies of the interactions of drugs and NPs at the transporter level have been limited so far, the in vivo and in vitro analyses presented here suggest that these interactions may be quite widespread, though they are currently underappreciated due to a severe lack of data compared to those available for transporter-level DDIs. Furthermore, the extent to which our results, mainly in mice, apply to humans remains to be clearly established. In this regard, it is worth noting that recent metabolomics studies in humans treated with probenecid indicate significant overlap with metabolomics data from Oat1 and Oat3 knockout mice [40].

## Data Availability

The original contributions presented in this study are included in the article/Supplementary Materials.

## Supplementary Materials

The following supporting information can be downloaded at: https://www.mdpi.com/article/10.3390/nu16142242/s1, Table S1: In vivo metabolomics of OAT1KO against WT serum matched to each of the 3 natural product databases used. Metabolon provided superpathway and subpathway designations are provided, in addition to *p*-value and fold change; Table S2: In vivo metabolomics of OAT3KO against WT serum matched to each of the 3 natural product databases used. Metabolon-provided superpathway and subpathway designations are provided, in addition to *p*-value and fold change; Table S3: OAT1 in vitro-associated compounds matched to at least one NP database; Table S4: OAT3 in vitro-associated compounds matched to at least one NP database. References [82,83,84,85,86,87,88,89] are cited in Supplementary Materials.
